# Methyl Viologen Lead Iodide for Photocatalytic Reductive Coupling of Aromatic Carbonyls via Proton-Coupled Electron Transfer

**DOI:** 10.1021/acsami.5c22726

**Published:** 2026-02-20

**Authors:** Minyang Yin, Ruichen Wan, Tsu-Hao Wang, Yiying Wu

**Affiliations:** Department of Chemistry and Biochemistry, 2647 The Ohio State University, 100 West 18th Avenue, Columbus, Ohio 43210, United States

**Keywords:** organic−inorganic hybrid composites, proton-coupled electron transfer, photosynthesis, pinacol coupling

## Abstract

Organic–inorganic metal halides (OIMHs) have emerged as promising photocatalysts for organic transformations due to their excellent optoelectronic properties. However, their instability, particularly under protic conditions, has limited their broader applications. Here, we report that a facilely prepared methyl viologen lead iodide (MVPb_2_I_6_) powder can efficiently catalyze the visible-light-driven reductive coupling of aromatic aldehydes and ketones in protic solvents. Remarkably, MVPb_2_I_6_ retains its structural integrity after photoreduction, highlighting its robustness. These results suggest that incorporating quaternary pyridinium cations into the OIMHs can enhance their stability and expand their applicability in photocatalytic organic synthesis.

## Graphical abstract

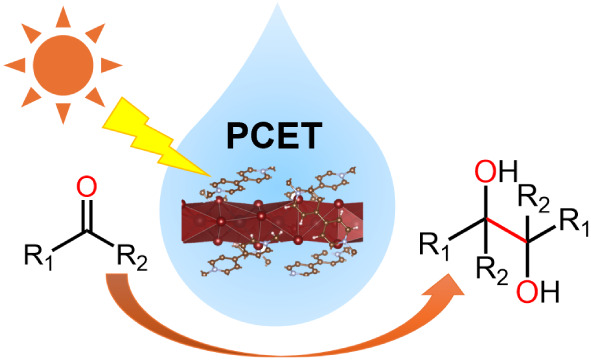

## Introduction

Harnessing solar energy for photocatalysis has long been of interest to the chemistry community.^1–3^ Following the pioneering work by MacMillan^4^ and Yoon,^5^ who demonstrated successful photodriven C–C bond formation using ruthenium-based photocatalysts, a widespread application of photocatalysts in the field of organic transformation has been witnessed in the last two decades.^6,7^ The design of photocatalysts plays a pivotal role in developing robust and reliable protocols for photoredox organic transformations. Various systems, including Ru/Ir-based metal complexes,^6,8–10^ organic dyes,^11,12^ and semiconductor quantum dots (QDs)^13,14^ etc., have been employed. However, these systems often require either noble metals or complex photocatalyst designs, which are less desirable for industrial applications.

In recent years, organic–inorganic metal halides (OIMHs) have attracted significant attention due to their remarkable optoelectronic properties, including high photoconversion efficiencies,^15^ long-range carrier migration,^16,17^ and prolonged excited-state lifetimes.^18,19^ These advantages have enabled the successful application of OIMHs in various optoelectronic applications, such as photovoltaics, photodetectors, transistors, laser-emitting diodes, and other optoelectrical devices.^20–24^ Notably, the properties that enhance optoelectrical applications are also expected to benefit photocatalytic applications.^25,26^ Compared to conventional semiconductor photocatalysts, the use of OIMHs offers simpler material preparation and greater tunability of the band structures through compositional control. Although still in its early stages, the use of OIMHs as photocatalysts for organic transformations has shown promising potential. Several reports have demonstrated that halide perovskite nanocrystals can effectively photocatalyze C–C, C–N, C–P, S–S, and C–O bond formations.^27–44^ However, a major challenge hindering the broader applications of OIMHs in photoredox organic transformation is their sensitivity to polar and protic solvents.^39,45,46^ This limitation not only constrains the choice of reaction media but also narrows the scope of accessible organic transformations.

Stability improvement for OIMHs has been pursued through heterojunctions, composite architectures, and double-perovskite platforms to enhance charge separation and mitigate degradation.^40,42,43^ Recently, several groups have reported that by exploiting the dynamic equilibrium between the dissolution and reprecipitation of MAPbI_3_, perovskite materials can exhibit considerable photocatalytic stability in aqueous solutions for hydrogen evolution reactions. However, such conditions typically require a highly acidic environment and a saturated concentration of primary ammonium salts, which are generally incompatible with most organic transformations.

An effective strategy to address these stability issues is using quaternary ammonium to construct OIMHs.^47–50^ Due to the full substitution on the N atom, quaternary ammoniums exhibit weak H-bonding donating and accepting ability. Therefore, 1D OIMHs constructed from quaternary ammonium cations generally exhibit superior stability in polar organic solvents and even in water.^50–52^ Compared to other 1D OIMHs, methyl viologen lead iodide (MVPb_2_I_6_) stands out as a potential photocatalyst due to its relatively small bandgap (∼2.1 eV). This property is attributed to the use of conjugated organic cations for charge-transfer excitation.^53^

Here, we report the first demonstration of an OIMH-based photocatalytic system utilizing MVPb_2_I_6_ powder for photoredox organic transformations under visible-light irradiation via a proton-coupled electron transfer (PCET) mechanism (Figure 1). We selected the pinacol coupling of aromatic carbonyls as a model reaction, given that the activation of aromatic carbonyls to generate ketyl radicals is a common method for C–C bond formation.^54–56^ However, the large mismatch between the reduction potentials of aromatic carbonyls (e.g., benzaldehyde: $E_{1/2}^{\mathrm{red}}=-1.93V\;\mathrm{vs}\;\mathrm{SCE}$ ; acetophenone: $E_{1/2}^{\mathrm{red}}=-2.11V\;\mathrm{vs}\;\mathrm{SCE}$ )^57,58^ and the conduction band edges of typical photocatalysts makes direct electron transfer highly endergonic. Established protocols typically involve a concerted PCET step to lower the activation barrier by either adding Brønsted acids or generating Brønsted-acidic α-ammonium radicals from the oxidation of amines within the system.^14,58,59^ Although the pinacol coupling of aromatic carbonyls is well established, conventional OIMH photocatalysts are typically incompatible with these protocols due to their instability under acidic conditions, which arises from acid–base equilibria involving ammonium cations within their structures. Therefore, to enable the use of the PCET mechanism with OIMHs, a new material design strategy was required. In this study, we demonstrate that our photocatalytic system can efficiently generate ketyl radicals from aromatic carbonyls via a PCET process. These radicals then underwent homocoupling to afford vicinal diols, as illustrated in Figure 1. Furthermore, MVPb_2_I_6_ photocatalyst exhibits excellent stability in protic media, positioning it as a promising candidate for a broader range of photoredox organic transformations.

**Figure 1:**
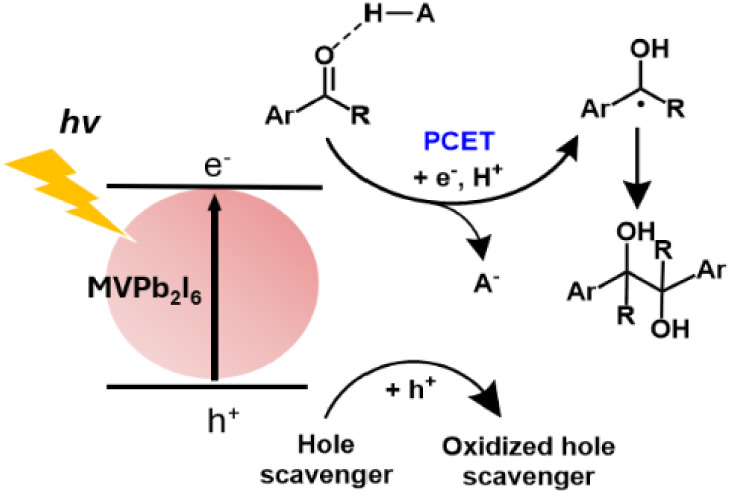
Postulated mechanism for MVPb2I6-catalyzed pinacol coupling of aromatic carbonyls through a PCET mechanism.

The PCET redox mechanism underpins energy conversion processes in biological systems,^60^ and facilitates the interconversion of important small molecules such as O_2_/H_2_O, N_2_/NH_3_ and CO_2_/alkane.^61–63^ The PCET process involves the concerted movement of electrons and protons in a single chemical step, offering both thermodynamic and kinetic advantages^64–66^ by avoiding high-energy intermediates typically formed in sequential electron transfer (ET) and proton transfer (PT) processes.^67^ Thus, PCET is also a general mechanism for photoredox organic transformations and has been utilized in various reactions.^4,62^ Therefore, our findings also highlight a novel perspective for employing OIMHs in organic transformations that have conventionally been overlooked or deemed incompatible.

## Methods

PbI_2_ (>99.99%) was purchased from TCI America. All other chemical reagents were purchased from Sigma-Aldrich. All solvents were used as received unless otherwise stated. All commercial reagents were used without further purification. Thin-layer chromatography (TLC) on silica gel plates (Select Scientific, 200 Micron, Cat. No. 31028, Silica Gel 60, F-254) was employed to monitor the reactions, visualized by ultraviolet (UV) light at a 254 nm wavelength. Flash column chromatography was performed on silica gel with a particle size of 40 to 63 microns. NMR spectra were recorded on a 400 MHz AVANCE III spectrometer and calibrated with residual chloroform (δ H = 7.26 ppm, δ C = 77.0 ppm) or dimethyl sulfoxide (δ H = 2.50 ppm, δ C = 39.5 ppm) as internal references. Chemical shifts were reported in ppm δ. The following abbreviations are used to indicate multiplicities: s = singlet, d = doublet, t = triplet, q = quartet, m = multiplet, br = broad.

A xenon lamp was used as the light source with an AM 1.5G filter for solar light simulation. The light intensity was calibrated to be one sun intensity (100 mW/cm²) by a power meter (Newport optical power meter) and a silicon photodiode (818-UV). Powder X-ray diffraction (PXRD) was recorded by a Bruker D8 ADVANCE X-ray diffractometer with a Cu Kα source (λ = 1.5406 Å), operated at 40 kV and 40 mA.
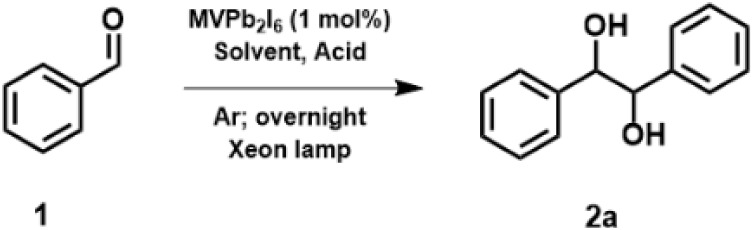


### Synthesis of Methyl Viologen Diiodide (MVI_2_)

2 mmol 4,4’-bipyridine and 4 mmol iodomethane were added to 10 mL of acetonitrile. The solution was stirred at 60 °C for 2 h to obtain an orange precipitate. After cooling, the crude product was collected by vacuum filtration and washed with acetonitrile. The collected orange solid was used directly without further purification.

### Synthesis of Methyl Viologen Lead (II) Iodide (MVPb_2_I_6_)

MVPb_2_I_6_ was synthesized by simply reacting PbI_2_ and MVI_2_ in solution. To be more specific, a precursor solution of PbI_2_ (1 mmol) was prepared in 2 mL of 57 wt % hydriodic acid, and MVI_2_ (0.5 mmol) was added to the solution under stirring. A maroon powder was collected and washed with diethyl ether three times by centrifugation. The powder samples were then dried under vacuum at 50 °C for further experiments. PXRD was conducted and compared with the reported CIF file to confirm the structure.

## Results and Discussions

The MVPb_2_I_6_ photocatalyst was synthesized following a reported procedure^68^ by simply reacting lead iodide (PbI_2_) and methyl viologen (MVI_2_) in solution. The resulting powder was used directly as a photocatalyst after rinsing and drying, without the need for surface passivation, which is typically required for other perovskite-type photocatalysts reported to date in photoredox organic transformations.^26,27,32^ The facile-prepared, modification-free features of MVPb_2_I_6_ further offer an example of a low-cost, user-friendly approach for photoredox organic transformations compared to other semiconductor-based protocols.

The crystal structure of MVPb_2_I_6_ was depicted in Figure 2, it consists of 1D chains of face-sharing [Pb–I_6/2_]^−^ octahedrons surrounded by MV^2+^ cations (plotted from its reported CIF file^68^). The conduction band and valence band were previously characterized to be −1.24 and 0.86 V vs SCE, respectively, according to previous reports.^53^

**Figure 2:**
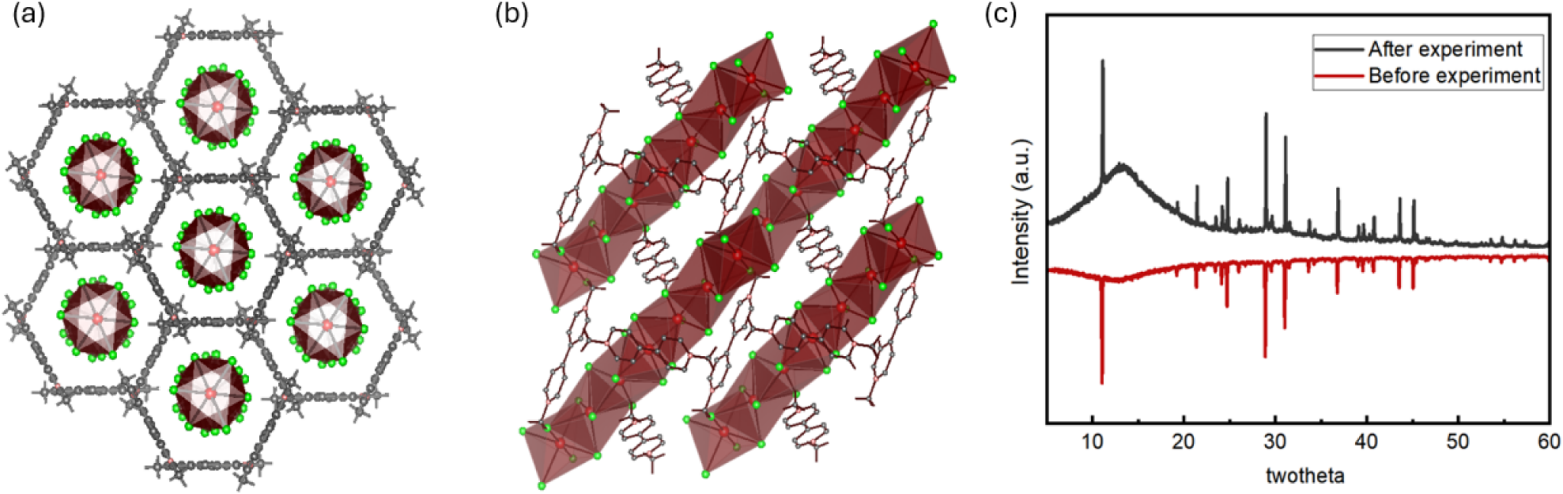
(a) Crystal structure of 1D MVPb_2_I_6_; (b) view of lead iodide quantum wire wrapped with MV^2+^ organic cations; and (c) PXRD of MVPb_2_I_6_ before (red) and after (gray) photocatalytic experiment for entry 6 in Table 1.

We commenced our investigation by exploring the photoreduction of benzaldehyde $\left(E_{1/2}^{\mathrm{red}}=-1.93V\;\mathrm{vs}\;\mathrm{SCE}\right)$ . Selected results are summarized in Table 1. We first tested the use of tertiary amines as hole scavengers in photocatalytic systems. Upon oxidation, the obtained amino radical cation would undergo a [1,2]-H shift to generate Brønsted-acidic α-ammonium radicals (illustrated in Figure S3), which would then initiate the PCET process through H-bonding to the C=O bond, in coordination with previous reports.^58^ Pinacol product was observed (Table S1, entries 2 and 3). ^1^H NMR spectral analysis was employed to determine the yield and diastereoselectivity (dl:meso ratio). However, while amines were present in the system, noticeable degradation of the photocatalyst occurred, as reaction solutions turned orange after irradiation (Figure S2a). Notably, MVPb_2_I_6_ was completely dissolved when acetonitrile (MeCN) was used as the solvent (Table S1, entry 1). Powder X-ray diffraction (PXRD) analysis was also conducted after irradiation, and new peaks were present (Figure S2b), which might be due to cation exchange between the methyl viologen cation and the α-ammonium radical. These findings suggest that tertiary amines are not suitable hole scavengers for our system.

**Table 1:** Optimization Studies of the Photocatalyzed Pinacol Coupling of Benzaldehyde­[^a^]

Entry	Solvent[^b^]	Acid[^c^]	Yield (%) [^d^]	meso:dl
1[^e^]	H_2_O	Acetate buffer	73	1:1.23
2	H_2_O	CH_3_COOH	54	1:1.21
3	MeCN	CH_3_COOH	n.r.	-
4	Toluene	CH_3_COOH	n.r.	-
5[^f^]	EtOAc/H_2_O	CH_3_COOH	14	1.05:1
6^g^	EtOAc/H_2_O	CH_3_COOH	85	1:1.2

a Reaction conditions: **1a** (1 mmol), MVPb_2_I_6_ (13.6 mg; 1 mol %), room temperature, Xeon lamp (350 W), 16 h.

b Degassed solvent, 4 mL.

c 10 mmol of acetic acid.

d Determined by NMR analysis. n.r. = no reaction.

e 1 mL of pH = 4 acetate buffer (0.1 M) was used instead of glacial acetic acid.

f EtOAc:H_2_O (v/v = 9:1) 4 mL.

g EtOAc:H2O (v/v = 3:1).

In Table 1, ethanol was employed as the hole scavenger, and no noticeable color change was observed after irradiation (Figure S2a), indicating the stability of the photocatalyst under these conditions. The diol product **2a** was observed only when water was present in the system, suggesting that water plays a critical role in facilitating the reaction. Since the proposed mechanism involves carbonyl activation via the PCET process, the acid’s p*K*_a_ is a critical factor. Notably, the p*K*_a_ of acetic acid is approximately 4.76 in water, but increases significantly in less polar solvents.^69^ This increase suppresses acetic acid dissociation, thereby reducing proton availability in these media. The acid concentration was found to affect not only the reaction yield but also product selectivity. In entry 2, where water was used as the sole solvent, both 1,2-diphenylethane-1,2-diol (the pinacol product) and benzyl alcohol (the competing product) were observed with yields of 54% and 27%, respectively. To improve substrate solubility, a mixture of ethyl acetate (EtOAc) and H_2_O (*v*/*v* = 3:1) was selected for subsequent experiments. Meanwhile, the PXRD patterns of MVPb_2_I_6_ exhibit no noticeable changes before and after the reaction, indicating that MVPb_2_I_6_ retained its structural integrity following photoreduction (Figure 2b). The stability of MVPb_2_I_6_ in protic media is consistent with the known stabilizing role of the quaternary ammonium cations. A primary failure mode of OIMHs constructed by primary ammonium salt is acid–base chemistry, which facilitates lattice decomposition; viologen cations, in contrast, are fully quaternized and thus do not undergo analogous acid–base equilibria with water.^52^ Indeed, viologen-based lead iodides have been reported to tolerate prolonged water exposure compared to primary ammonium analogues.^49,52,70^ In addition to stability under photocatalytic conditions, we also investigated the compatibility of MVPb_2_I_6_ with a range of common solvents such as ethanol, DCM, hexane, toluene, acetone, MeCN, IPA, and ethyl acetate by ^1^H NMR and PXRD analyses (Figures S5–S15, Supporting Information). The crystalline phase was retained, whereas partial dissolution was observed in strongly coordinating solvents such as DMF and DMSO. These results further highlight the robustness of MVPb_2_I_6_ in protic media and rationalize its unique applicability in photoredox organic transformations.

Furthermore, the recyclability of MVPb_2_I_6_ was evaluated through scaled-up catalytic runs (200 mg under standard conditions, proportionally scaled). The catalyst was subjected to three consecutive photocatalytic cycles, affording consistent activity and structural integrity. After the first cycle, the recovered powder weighed 193 mg (96.5% recovery), which we attribute to inevitable handling losses. The PXRD patterns of the recycled samples remained identical to the pristine material, with only minor intensity variations, confirming the robustness of MVPb_2_I_6_ as a recyclable heterogeneous photocatalyst.

The reproducibility of the reaction was also validated through repeated experiments, and a series of control experiments were conducted (Table S2, entries 2–6). These results highlighted the essential interplay between light irradiation, ethanol, the photocatalyst, acetic acid, and oxygen exclusion in achieving the desired photoredox organic transformation.

After establishing the optimized reaction conditions, we investigated the feasibility of extending our system to ketone substrates, specifically acetophenone $\left(E_{1/2}^{\mathrm{red}}=-2.11V\;\mathrm{vs}\;\mathrm{SCE}\right)$ and benzophenone $\left(E_{1/2}^{\mathrm{red}}=-1.52V\;\mathrm{vs}\;\mathrm{SCE}\right)$ .^58^ It was observed that benzophenone readily underwent conversion to the corresponding diols in high yield without further modification. In contrast, acetophenone showed no reactivity, even when acetic acid was replaced with trifluoroacetic acid (p*K*_a_ = 0.52 in water),^71^ a significantly stronger Brønsted acid. This lack of reactivity is likely due to the reduction potential of acetophenone exceeding the reducing capacity of MVPb_2_I_6_. We further accessed the scope of this protocol, and the results are summarized in Table 2. The presence of electron-withdrawing and -donating groups was found to significantly influence the reaction yield. While acetophenone itself remained unreactive, substrates bearing electron-withdrawing groups, such as −F and −CF_3_, facilitated successful pinacol coupling. In contrast, substrates containing electron-donating groups such as −OH, −OCH_3_, or −SCH_3_ were found to be inert to the protocol, even for the aromatic aldehydes. Substituents influence reactivity and yields by shifting the carbonyl reduction potential. Electron-withdrawing groups lower the carbonyl π* (LUMO) energy and shift the first reduction to more positive potentials, whereas electron-donating groups raise the π* (LUMO) energy and shift the reduction to more negative values; consequently, ketyl intermediate formation is slower, and back electron transfer/recombination becomes more competitive under identical irradiation conditions, leading to diminished isolated yields. This limited applicability primarily arises from the relatively low reducing power of MVPb_2_I_6_, confining the protocol to a narrow scope of aromatic carbonyl compounds.

**Table 2:**
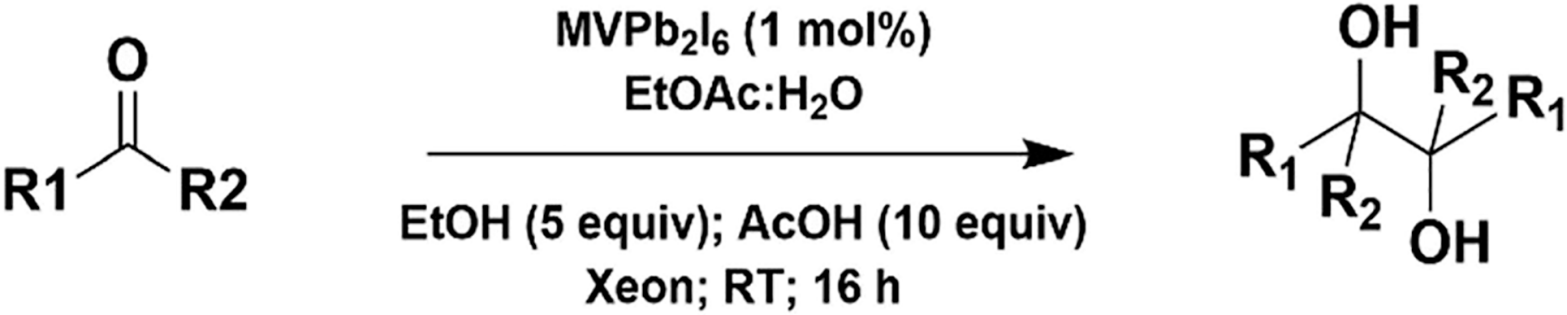

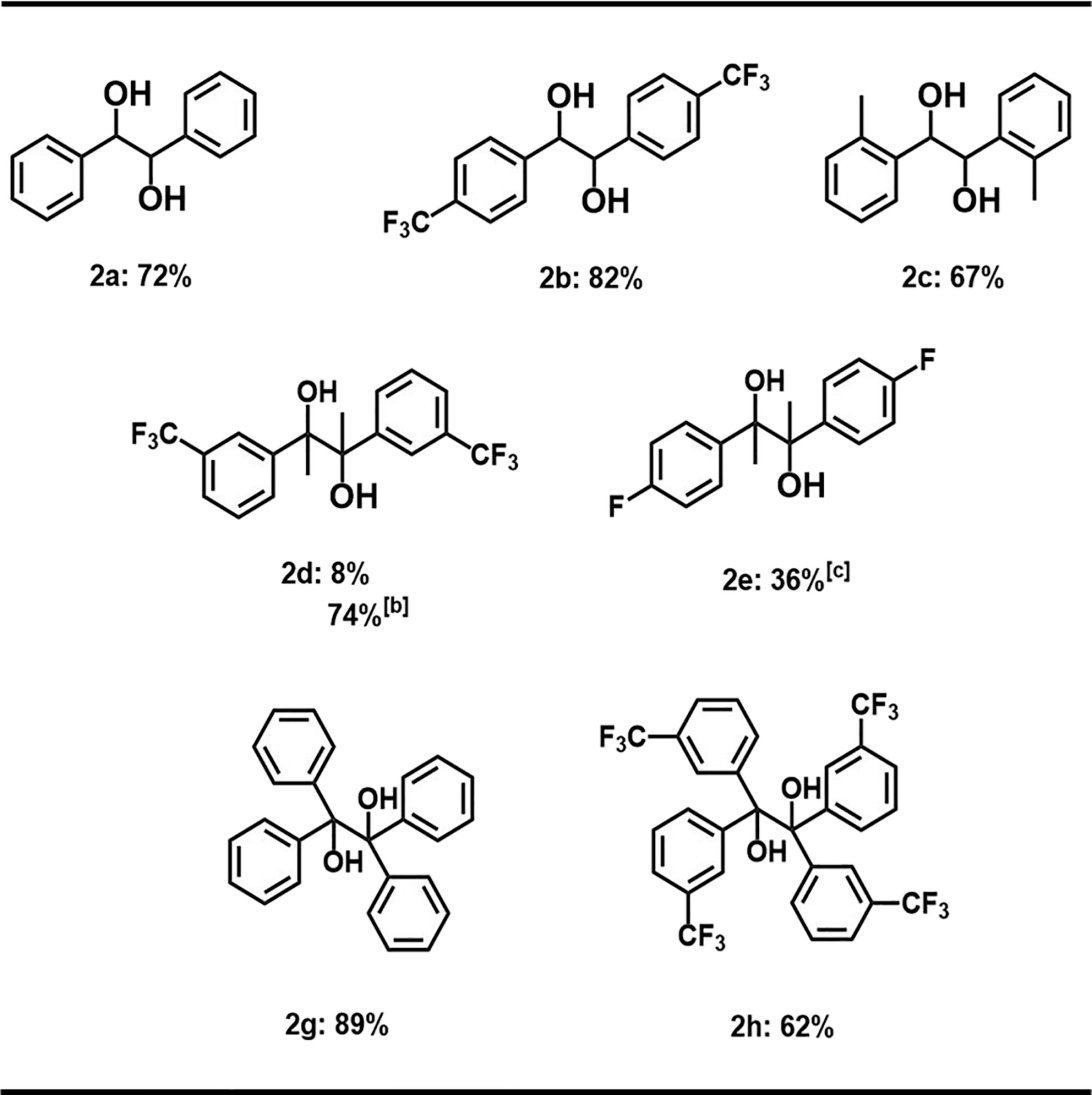
Scope of Photocatalytic Pinacol Coupling of Aromatic Carbonyls­[^a^]

a Reaction conditions: aromatic carbonyls (1 mmol), EtOAc:H_2_O (v/v = 3:1) 4 mL, MVPb_2_I_6_ (1 mol %), EtOH (5 equiv.), AcOH (10 equiv.), Ar, RT, Xeon lamp, 16 h.

b Trifluoroacetic acid (10 equiv.) was used instead of acetic acid.

c 1 mL pH = 4 acetate buffer (0.1 M) was used instead of glacial acetic acid.

We also observed remarkable tunability in product selectivity when the substrates contained a pyridine moiety. As shown in Scheme 1, under the established protocol, the reaction predominantly yielded alcohol as the final product. However, when the Brønsted acid was replaced with 1 mL of pH 4 acetate buffer (0.1 M), the diol product was obtained solely. This difference in selectivity suggests the involvement of an alternative pathway during the photoreduction of pyridine-containing substrates. This marked difference in selectivity suggests the involvement of an alternative reaction pathway during the photoreduction of pyridine-containing substrates, as shown in Scheme 2. Specifically, in the presence of acetate buffer, the majority of 4-acetylpyridine remains unprotonated and undergoes a one-electron–one-proton proton-coupled electron transfer (PCET) process to generate the ketyl radical (**A**), as supported by a TEMPO radical trapping experiment (see Supporting Information, Figure S4), Conversely, under sufficiently acidic conditions, the predominant species is the protonated pyridinium ion (**1f/i-H**), which undergoes a two-electron–one-proton reduction to form a relatively stable enol intermediate (**B**). This intermediate subsequently undergoes protonation to yield the corresponding alcohol, as depicted in Scheme 2. These observations are consistent with previous reports on the electroreduction of 4-acetylpyridine under acidic conditions.^72–74^ To further substantiate our mechanistic rationale, we directly monitored the product distribution of 4-acetylpyridine under varying acetic acid concentrations by ^1H NMR spectroscopy (Figure S16 and Supporting Information). A monotonic shift was observed between the diagnostic pinacol OH resonances (δ 5.3–5.5 ppm) and the alcohol quartet (δ ∼ 4.9 ppm), providing clear spectroscopic evidence that proton availability governs the reaction pathway. This observation is fully consistent with established proton-coupled electron transfer (PCET) mechanisms for ketyl radical chemistry. Notably, prior electrochemical studies by Köster and coworkers^72^ demonstrated a formal second-order dependence of the 4-acetylpyridine reduction on proton concentration and proposed a stepwise sequence of (i) rapid protonation, (ii) concerted two-electron/one-proton reduction to a stabilized carbanion, and (iii) subsequent protonation to yield the alcohol. We therefore infer that the photoelectrochemical pathway under our conditions follows an analogous PCET-controlled process, in line with both our spectroscopic evidence and authoritative literature precedents.

**Scheme 1:**
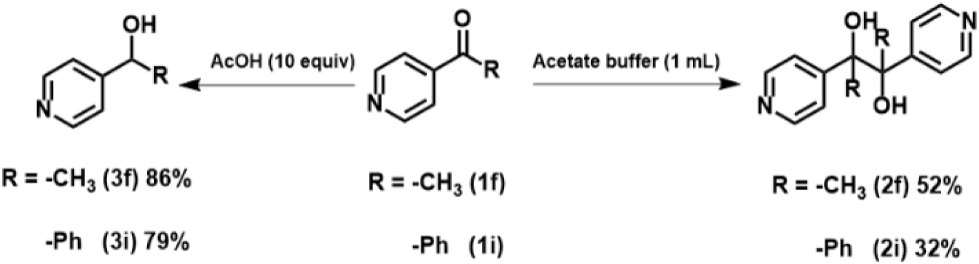
Product Tunability Under Different pH Conditions for Pyridyl Ketone Substrates

**Scheme 2:**
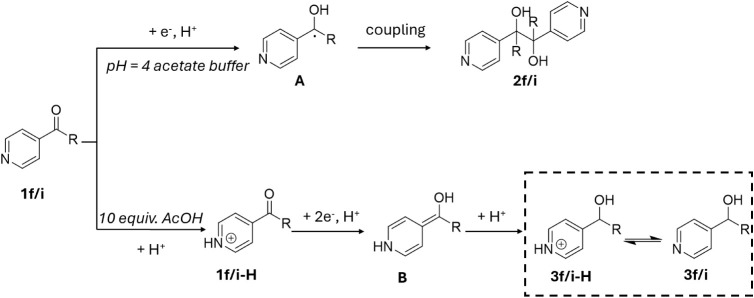
Photocatalytic Pathways for Photoreduction of Pyridyl Ketone Substrates Uunder Different pH Environment

## Conclusions

In conclusion, we developed a photocatalytic system based on OIMHs that leverages a PCET mechanism during photocatalysis, representing a novel strategy for photoredox organic transformations enabled by OIMHs. The photocatalyst MVPb_2_I_6_, obtained by facile preparation, demonstrated the ability to effectively promote the pinacol coupling of aromatic carbonyls in the presence of Brønsted acids. The incorporation of quaternary ammonium cations into the OIMH structure proved to be an effective strategy in improving stability under protic conditions, which traditionally limit the use of OIMHs in photocatalysis. The MVPb_2_I_6_ photocatalyst maintained its structural integrity throughout the reactions, highlighting its robustness and potential for broader applications. While the reaction scope is limited by the relatively low reducing power of MVPb_2_I_6_, the inherent tunability of the OIMHs suggests that the photocatalytic capability could be enhanced through rational compositional design.

Our findings demonstrate that employing a PCET strategy with OIMHs can expand their application scope in photoredox organic transformations, particularly in environments previously considered challenging due to the stability concerns of conventional OIMHs. Moreover, despite the relatively low photoreducing power of MVPb_2_I_6_, PCET allows it to access reactions that are not accessible without proton transfer. Future efforts should focus on optimizing the band structure of OIMHs by rational compositional design, potentially broadening the range of accessible photoredox organic transformations. In addition, the presence of Pb raises environmental and health concerns, motivating future work on lead-leaching quantification under operational conditions, immobilization/encapsulation strategies to mitigate release, and the development of lead-free OIMH analogues. Finally, although MVPb_2_I_6_ retains crystallinity over the reaction times examined here, systematic long-duration irradiation studies (activity decay, structural evolution, and leaching analyses) are important to establish durability under extended operation.

## Supplementary Material



## Notes

Experimental details are included in the Supporting Information (PDF)
