# Supplementary material for: Methyl Viologen Lead Iodide for Photocatalytic Reductive Coupling of Aromatic Carbonyls via Proton-Coupled Electron Transfer
Source: ACS Appl Mater Interfaces. 2026 Feb 20;18(8):12643–9. doi: 10.1021/acsami.5c22726 (PMC12964341; doi:10.1021/acsami.5c22726)
Supplement: Supplementary file 1 [file am5c22726_si_001.pdf]

Supporting information

Methyl Viologen Lead Iodide for Photocatalytic Reductive Coupling of Aromatic Carbonyls via Proton-Coupled Electron Transfer

Minyang Yin, Ruichen Wan, Tsu-Hao Wang, and Yiying Wu*

Department of Chemistry and Biochemistry, The Ohio State University, 100 West 18th Avenue, Columbus, OH 43210, United States

E-mail: wu@chemistry.ohio-state.edu

1. General Experimental Methods

PbI_2 (>99.99%) was purchased from TCI America. All other chemical reagents were purchased from Sigma-Aldrich. All solvents were used as received unless otherwise stated. All commercial reagents were used without further purification. Thin-layer chromatography (TLC) on silica gel plates (Select Scientific, 200 Micron, Cat no. 31028, Silica gel 60, F-254) were employed to monitor the reactions visualized by ultraviolent (UV) light at 254 nm wavelength. Flash column chromatography was performed on silica gel from 40 to 63 Micron. NMR spectra were recorded on 400 MHz AVANCE III spectrometer and calibrated with residual chloroform (δ H = 7.26 ppm, δ C = 77.0 ppm) or dimethyl sulfoxide (δ H = 2.50 ppm, δ C = 39.5 ppm) as internal references. Chemical shifts were reported in ppm δ. The following abbreviations are used to indicate multiplicities: s = singlet, d = doublet, t = triplet, q = quartet, m = multiplet, br = broad.

A Xenon lamp was applied as the light source with an AM 1.5G filter for solar light simulation. The light intensity was calibrated to be one sun intensity (100 mW/cm^2) by a power meter (Newport optical power meter) and a silicon photodiode (818-UV). Powder X-ray diffraction (PXRD) was recorded by a Bruker D8 ADVANCE X-ray diffractometer with a Cu Kα source (λ = 1.5406 Å) and operated at 40 kV and 40 mA.

2. Preparation of Methyl Viologen Lead Iodide ($MVPb_2I_6$)

Synthesis of Methyl Viologen Diiodide (MVI_2): MVI_2 was synthesized following literature procedure. [1] 2 mmol 4,4'-bipyridine and 4 mmol iodomethane were added to 10 mL of acetonitrile. The solution was stirred under 60 °C for 2 h to obtain an orange precipitate. After cooling down, the crude product was collected by vacuum filtration and washed with acetonitrile. The collected orange solid was used directly without further purification.

Synthesis of Methyl Viologen Lead (II) Iodide ($MVPb_2I_6$): $MVPb_2I_6$ was synthesized following a reported procedure by simply reacting PbI_2 and MVI_2 in solution. [2] Be more specific, a precursor solution of PbI_2 (1 mmol) was prepared in 2 mL 57 wt% hydriodic acid, and MVI_2 (0.5 mmol) was added to the solution under stirring. Maroon powder was collected and washed with diethyl ether three times by centrifugation. The powder samples were then dried under vacuum at 50 °C for further experiments. [3] PXRD was conducted and compared with reported CIF file to confirm the structure.

3. Full Optimization Studies for the Pinacol Coupling of Benzaldehyde

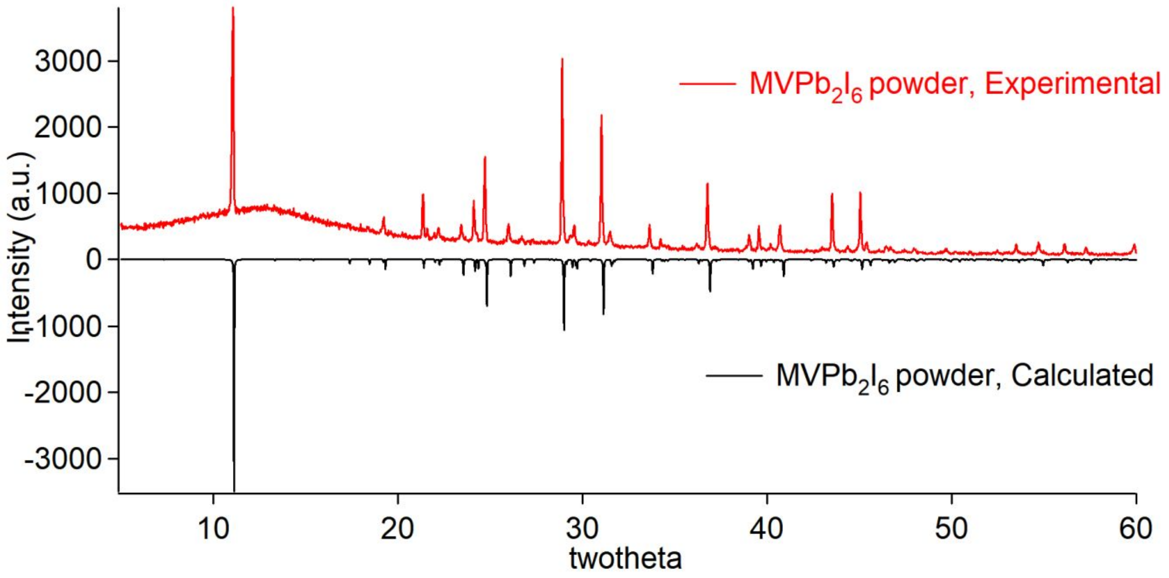

Figure S1. PXRD of $MVPb_2I_6$ powder, the red curve is experimental pattern, while the black curve is the calculated one.

Table S1. Utilizing Amine as Sacrificer.[a]

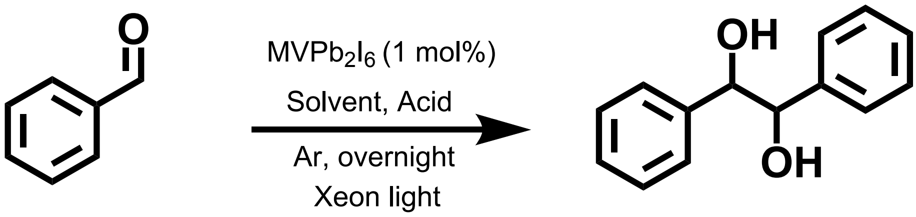

1a **2a**

Entry	Solvent	Sacrificer[b]	Yield(%)[c]	dl:meso
1	MeCN	Et_3N	n.r.	-
2	MeCN	DIPEA	16	1:1.25
3	Toluene	Et_3N	53	1:1.5

[a] Reaction condition: benzaldehyde 1a (1 mmol), $MVPb_2I_6$ (13.6 mg; 1 mol%), RT, solvent 4 mL degassed; Xeon lamp (350 W), 16 h. [b] 5 mmol of sacrificer. [c]: Determined by ^1H NMR analysis. n.r. = no reaction.

Table S2. Control Experiments.[a]

Entry	Deviation from the standard conditions	Yield (%)	dl:meso
1	-	85	1:1.2
2	No acid	n.r	-
3	No degassing	n.r	-

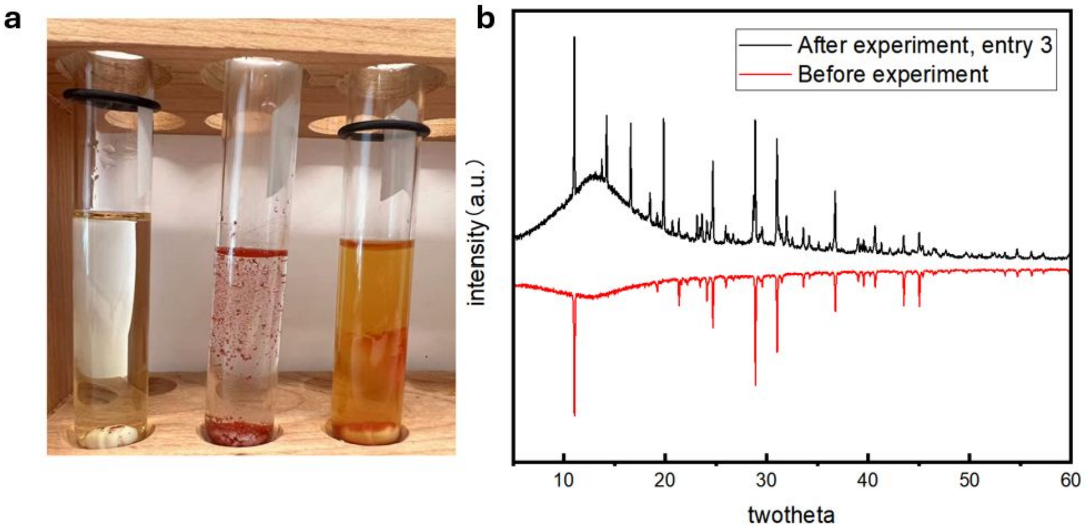

Figure S2. a) From left to right, reaction solutions of entry **6** (table 1), **2** (table 1) and **3** (table S1) correspondingly. Colored solution from entry 3 indicates possible corrosions of tertiary amine to the photocatalyst. b) PXRD of $MVPb_2I_6$ before (red) and after (grey) experiment for entry **3** (table S1). Extra peaks are observed after experiments, indicating changes in the crystal structure of $MVPb_2I_6$ during photocatalysis.

5. Proposed Mechanism under Tertiary Amine

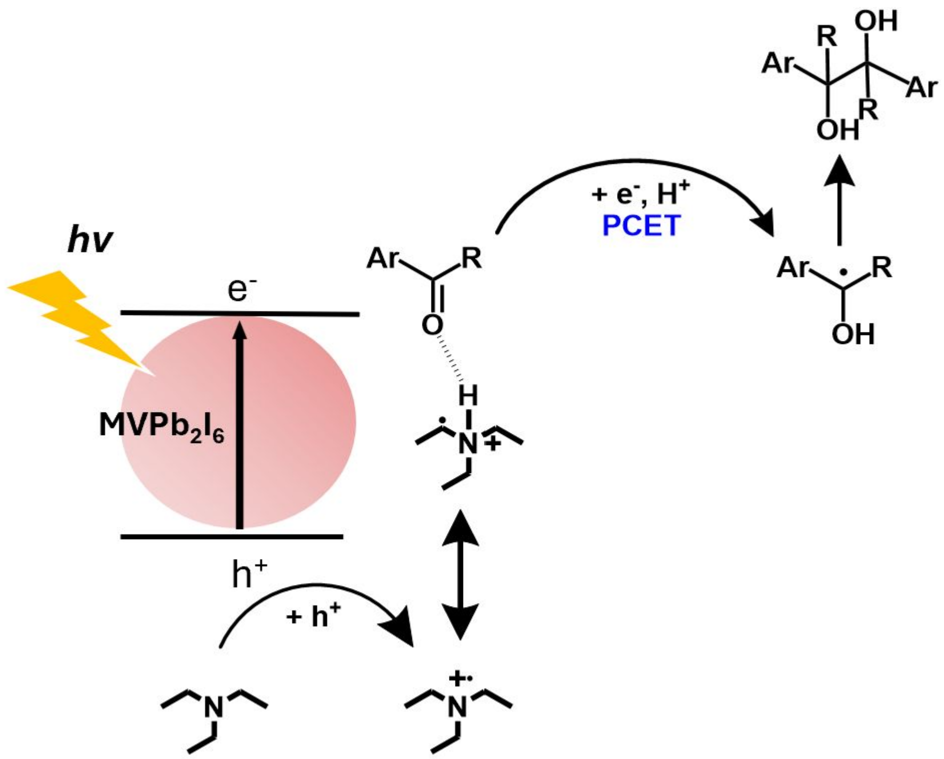

Figure S3. Proposed mechanism for tertiary amine promoted pinacol coupling of aromatic carbonyls.

6. Radical Trapping Experiment

In an air-tight vial charged with 4-acetylpyridine (1 mmol), $MVPb_2I_6$ (13.6 mg 1 mol%), ethanol (5 mmol), acetate buffer (0.1 M, 1 mL), TEMPO (1.5 mmol,1.5 equiv), 3 mL ethyl acetate and 1 mL water. Then the vial was degassed by argon for 5 minutes. The vial was irradiated by Xeon lamp at room temperature for 16 hours. The reaction mixture was centrifuged to remove $MVPb_2I_6$ first, the solution was neutralized by potassium bicarbonate and extracted by ethyl acetate. The combined organic layers were washed with Brine, dried over $MgSO_4$ and filtered. Purification of the crude product by column chromatography to afford the TEMPO trapped compound **1f-TEMPO**, which is confirmed by ^{1}H NMR (**Figure S4**).

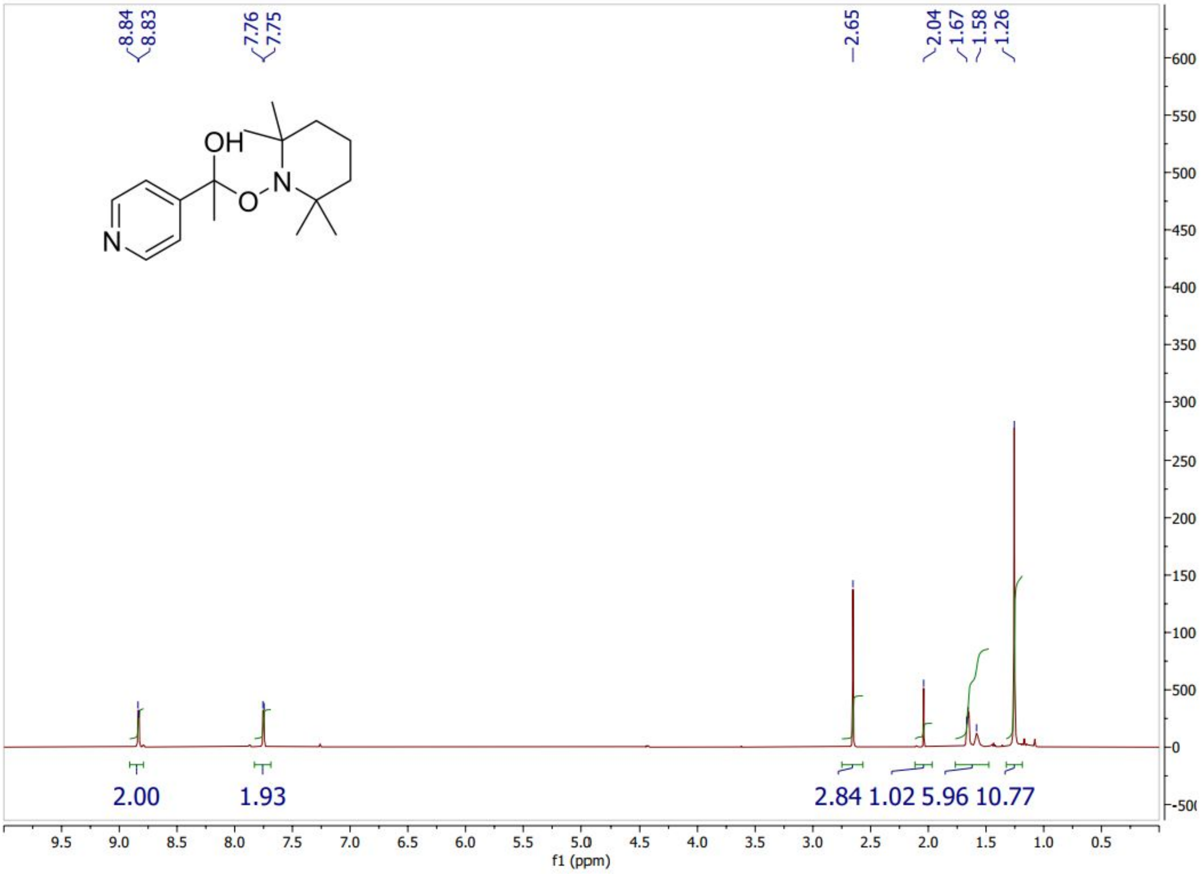

Figure S4. ^{1}H NMR for **1f-TEMPO.**

7. General procedure for the reductive dimerization of aldehydes and ketones

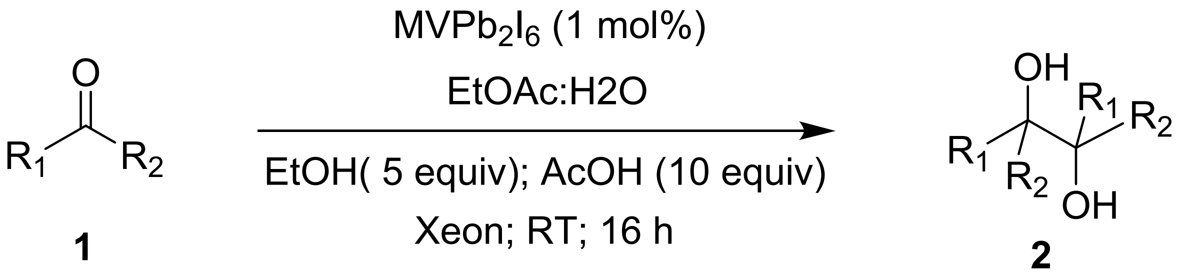

An air-tight vial was charged with aromatic carbonyls **1** (1 mmol), $MVPb_2I_6$ (13.6 mg 1 mol%), ethanol (5 mmol), acetic acid (10 mmol), 3 mL ethyl acetate and 1 mL water.

Then the vial was degassed by argon for 5 minutes. The vial was irradiated by Xeon lamp at room temperature for 16 hours. The reaction mixture was centrifuged to remove $MVPb_2I_6$ first, the solution was neutralized by potassium bicarbonate and extracted by ethyl acetate. The combined organic layers were washed with Brine, dried over $MgSO_4$ and filtered. The product was obtained by a short plug of silica gel.

1,2-diphenylethane-1,2-diol (2a): The titled product was synthesized according to the general procedure employing benzaldehyde **1a** (1 mmol), ethanol (5 mmol) and acetic acid (10 mmol). The product was purified by flash column chromatography. **Yield** 72%; **meso:dl** 1: 1.2; ^{1}H NMR of (dl)- and (meso)-**2a** (400 MHz, $CDCl_3$) δ 7.35 - 7.28 (m, 2.2 × 3H), 7.26 – 7.21 (m, 2.2 × 5H), 7.14 – 7.10 (m, 2.2 × 2H), 4.85 (s, 2H, meso), 4.72 (s, 1.2 × 2H, dl); ^{13}C NMR of (dl)- and (meso)-**2a** (100 MHz, $CDCl_3$) δ 139.8, 139.7, 128.2, 128.1, 127.9, 127.1, 126.9, 79.1, 78.1. The analytical data is in accordance with those reported in the literature. [4]

1,2-Bis(4-(trifluoromethyl)phenyl)ethane-1,2-diol (2b): The titled product was synthesized according to the general procedure employing 4-(trifluoromethyl)benzaldehyde **1b** (1 mmol), ethanol (5 mmol) and acetic acid (10 mmol). The product was purified by flash column chromatography. **Yield** 82%; **meso:dl** 1: 1; ^{1}H-NMR of (dl)- and (meso)-**2b** (400 MHz, $CDCl_3$) δ 7.56 (m, J = 8.0 Hz, 8H), 7.32 (d, J = 6.9 Hz, 4H), 7.25 (d, J = 8.0 Hz, 4H), 4.99 (s, 2H, meso), 4.77 (s, 2H, dl); ^{13}C NMR of (dl)- and (meso)-**2b** (100 MHz, $CDCl_3$) δ 145.3, 142.1, 131.3, 130.9, 127.1, 127.3, 124.8, 124.0, 122.2, 122.0, 119.1, 78.3, 77.1. The analytical data is in accordance with those reported in the literature. [5]

1,2-di-o-tolylethane-1,2-diol (2c): The titled product was synthesized according to the general procedure employing 2-methylbenzaldehyde **1c** (1 mmol), ethanol (5 mmol) and acetic acid (10 mmol). The product was purified by flash column chromatography. **Yield** 67%; **meso:dl** 1: 1.15; ^{1}H NMR of (dl)- and (meso)-**2c** (400 MHz, $CDCl_3$) δ 7.62 (d, J = 7.60 Hz, 1.15 × 2H), 7.33 (dd, J = 5.5, 3.7 Hz, 2H), 7.23 – 7.21 (m, 1.15 × 2H), 7.20 – 7.16 (m, 4H), 7.13 (td, J = 7.6, 1.3 Hz, 1.15 × 2Hz), 7.08 (dd, J = 5.5, 3.7 Hz),

6.91 (d, J = 7.6 Hz, 1.15 × 2H), 5.20 (s, 2H, meso), 4.98 (s, 1.15 × 2H, dl), 2.18 (s, 6H, meso), 1.67 (s, 1.15 × 6H, dl); ^{13}C NMR of (dl)- and (meso)- **2c** (100 MHz, $CDCl_3$) δ 139.1, 138.9, 136.1, 126.0, 130.4, 130.2, 129.1, 128.4, 126.8, 126.2, 126.0, 75.8, 74.6, 20.2, 19.8. The analytical data is in accordance with those reported in the literature. [4]

2,3-bis(3-(trifluoromethyl)phenyl)butane-2,3-diol (2d): The titled product was synthesized according to the general procedure employing 1-(3-(trifluoromethyl)phenyl)ethan-1-one **1d** (1 mmol), ethanol (5 mmol) and trifluoroacetic acid (10 mmol). The product was purified by flash column chromatography. **Yield** 74%; **meso:dl** 1: 1.2; ^1H NMR of (dl)- and (meso)-**2d** (400 MHz, $CDCl_3$) δ 7.55 – 7.40 (m, 6H), 7.40 – 7.28 (m, 10H), 1.61 (s, 6H, meso), 1.54 (s, 1.2 × 6H, dl); ^{13}C NMR of (dl)- and (meso)- **2d** (100 MHz, $CDCl_3$) δ 142.6, 142.4, 128.7, 128.2, 127.6, 127.3, 125.7, 125.4, 123.5, 123.2, 122.0, 122.1, 122.7, 122.7, 121.6, 120.6, 120.5, 76.3, 76.1, 22.6, 22.3. The analytical data is in accordance with those reported in the literature. [5]

2,3-bis(4-fluorophenyl)butane-2,3-diol (2e): The titled product was synthesized according to the general procedure employing 1-(4-fluorophenyl)ethan-1-one **1e** (1 mmol), ethanol (5 mmol) and acetic acid (10 mmol). The product was purified by flash column chromatography. **Yield** 736%; **meso:dl** 1: 1.1; ^1H NMR of (dl)- and (meso)-**2e** (400 MHz, $CDCl_3$) δ 7.20 – 7.16 (m, 2H), 7.415 – 7.10 (m, 2H), 6.95 – 6.88 (m, 4H)1.57 (s, 6H, meso), 1.49 (s, 1.1 × 6H, dl); ^{13}C NMR of (dl)- and (meso)- **2e** (100 MHz, $CDCl_3$) δ 142.6, 142.4, 128.7, 128.2, 127.6, 127.3, 125.7, 125.4, 123.5, 123.2, 122.0, 122.1, 122.7, 122.7, 121.6, 120.6, 120.5, 76.3, 76.1, 22.6, 22.3. The analytical data is in accordance with those reported in the literature.[6]

2,3-di(pyridin-4-yl)butane-2,3-diol (2f): The titled product was synthesized according to the general procedure employing 4-acetylpyridine **1f** (1 mmol), ethanol (5 mmol) and 0.1 M acetate buffer (1 mL). The product was filtered after neutralization. **Yield** 52%; ^1H NMR of **2f** (400 MHz, DMSO) δ 8.49 (dd, 4H), 7.52 (dd, 4H), 5.42 (s,

2H)1.23 (s, 6H); ^{13}C NMR of **2f** (100 MHz, DMSO) δ 156.2, 149.5, 120.8, 67.0, 25.3. The analytical data is in accordance with those reported in the literature. [7]

1,1,2,2-tetraphenylethane-1,2-diol (2g): The titled product was synthesized according to the general procedure employing benzophenone **1g** (1 mmol), ethanol (5 mmol) and acetic acid (10 mmol). The product was purified by flash column chromatography. **Yield** 89%; ^{1}H NMR of **2g** (400 MHz, $CDCl_3$) δ 7.32 – 7.25 (m, 8H), 7.20 – 7.06 (m, 12H), 3.36 (br, 2H); ^{13}C NMR of **2g** (100 MHz, $CDCl_3$) δ 143.8, 128.2, 127.2, 126.6, 82.8, 77.2. The analytical data is in accordance with those reported in the literature. [4]

1,1,2,2-tetrakis(3-(trifluoromethyl)phenyl)ethane-1,2-diol (2h): The titled product was synthesized according to the general procedure employing bis(3-(trifluoromethyl)phenyl)methanone **1h** (1 mmol), ethanol (5 mmol) and acetic acid (10 mmol). The product was purified by flash column chromatography. **Yield** 89%; ^{1}H NMR of **2h** (400 MHz, $CDCl_3$) δ 7.57 – 7.47 (m, 12H), 7.38 – 7.32 (t, J = 7.6 Hz, 4H), 3.02 (br, 2H); ^{13}C NMR of **2h** (100 MHz, $CDCl_3$) δ 143.8, 131.8, 130.4, 128.2, 125.2, 124.9, 124.5, 83.0.

1,2-diphenyl-1,2-di(pyridin-4-yl)ethane-1,2-diol (2i): The titled product was synthesized according to the general procedure employing phenyl(pyridin-4-yl)methanone **1i** (1 mmol), ethanol (5 mmol) and 0.1 M acetate buffer (1 mL). The product was filtered after neutralization. **Yield** 32%; ^{1}H NMR of **2i** (400 MHz, $CDCl_3$) δ 8.55 (d, J = 5.8 Hz, 2H), 7.40 – 7.30 (m, 12H), 3.35 (br, 2H); ^{13}C NMR of **2i** (100 MHz, $CDCl_3$) δ 148.9, 142.1, 128.9, 128.7, 128.5, 126.8, 121.5, 75.1.

1-(pyridin-4-yl)ethan-1-ol (3f): The titled product was synthesized according to the general procedure employing 4-acetylpyridine **1f** (1 mmol), ethanol (5 mmol) and acetic acid (10 mmol). The product was purified by flash column chromatography. **Yield** 86%; ^{1}H NMR of **3f** (400 MHz, $CDCl_3$) δ 8.49 (dd, J = 6.3, 2.8 Hz, 2H), 7.32 (dd,

J = 6.8, 2.9 Hz, 2H), 4.89 (q, J = 6.3 Hz, 1H), 1.48 (d, J = 6.6 Hz, 3H); ^{13}C NMR of **3f** (100 MHz, $CDCl_3$) δ 151.2, 132.1, 126.1, 118.5, 110.4, 69.0, 25.1. The analytical data is in accordance with those reported in the literature. [8]

phenyl(pyridin-4-yl)methanol (3i): The titled product was synthesized according to the general procedure employing phenyl(pyridin-4-yl)methanone **1i** (1 mmol), ethanol (5 mmol) and acetic acid (10 mmol). The product was purified by flash column chromatography. **Yield** 79%; ^{1}H NMR of **3i** (400 MHz, $CDCl_3$) δ 8.34 (dd, J = 6.3, 2.8 Hz, 2H), 7.34 – 7.24 (m, 7H), 5.75 (s, 1H), 4.84 (br, 1H); ^{13}C NMR of **3i** (100 MHz, $CDCl_3$) δ 153.1 149.0, 142.8, 128.5, 127.7, 126.8, 121.1, 74.2 The analytical data is in accordance with those reported in the literature. [9]

8. Stability test of $MVPb_2I_6$. in different solvents.

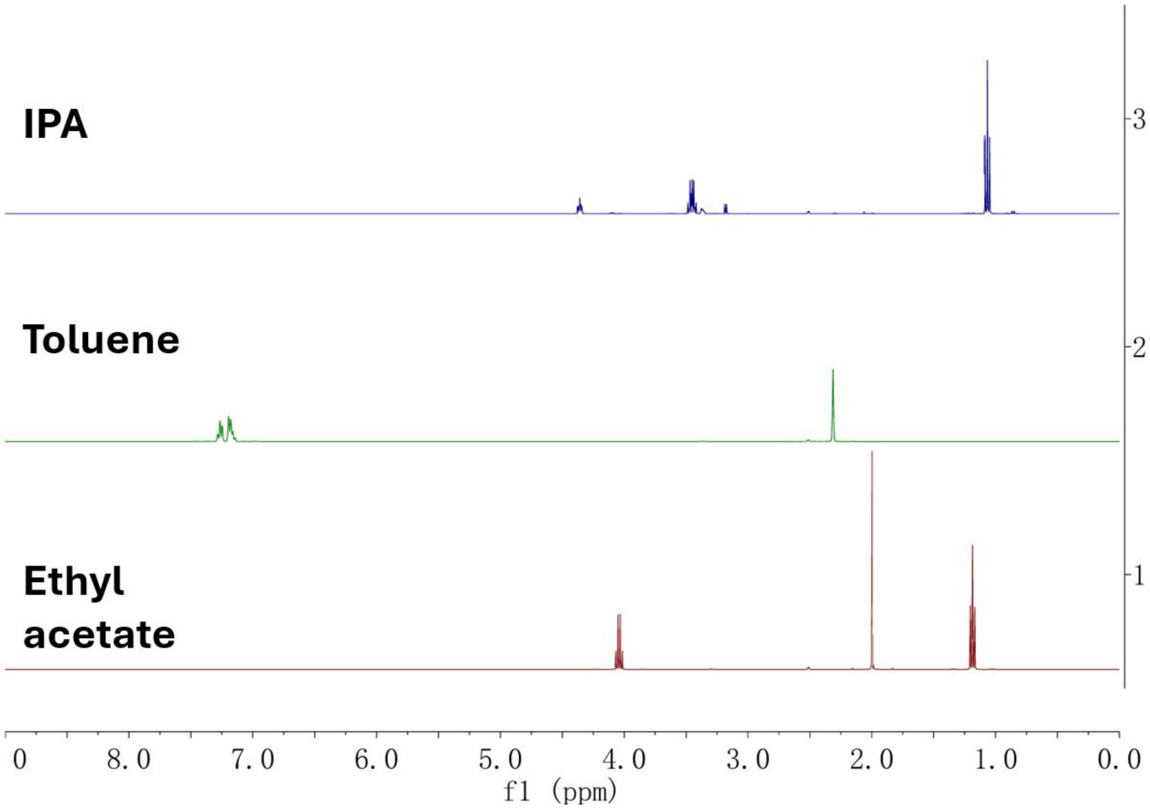

Figure S5. Solvent-stability test of $MVPb_2I_6$. The supernatant was extracted and added to DMSO-d_6 to run ^1H NMR of the supernatant after contacting $MVPb_2I_6$ with IPA, toluene, and ethyl acetate. There is no measurable leaching of MV^{2+} into solvents under these conditions.

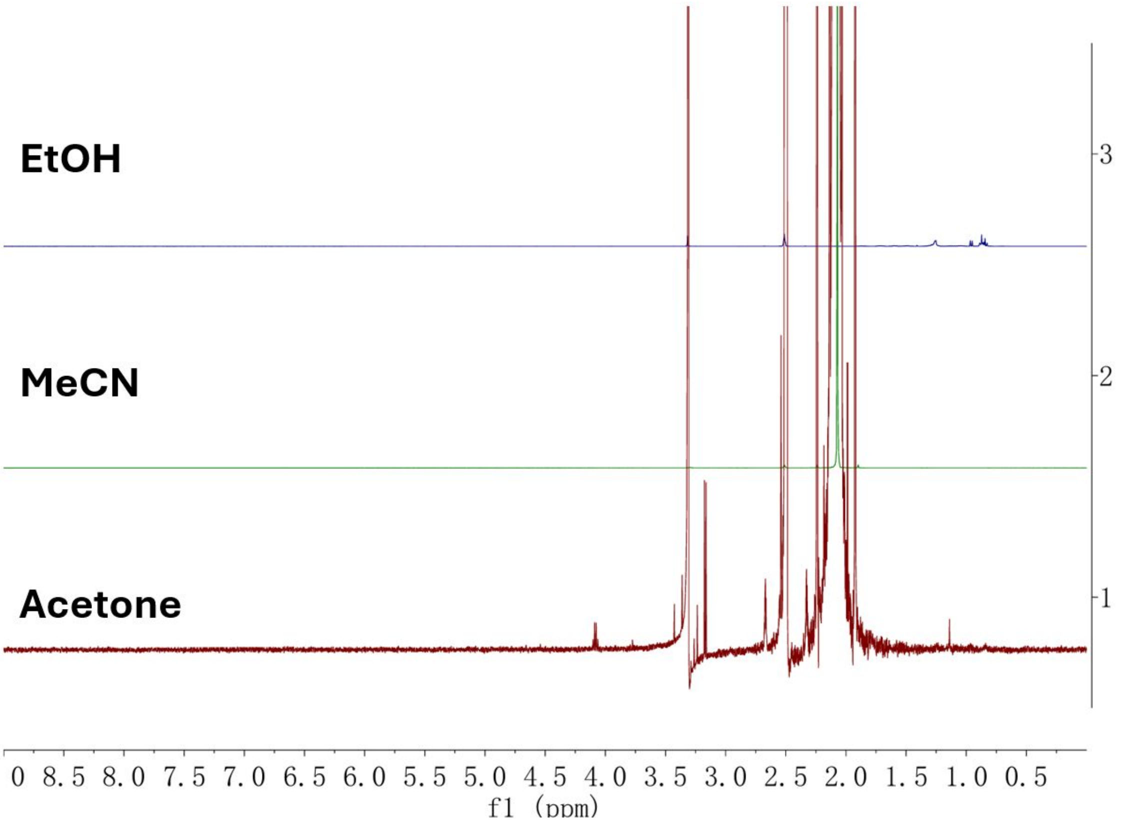

Figure S6. Solvent-stability test of $MVPb_2I_6$. The supernatant was extracted and added to DMSO-d_6 to run ^1H NMR of the supernatant after contacting $MVPb_2I_6$ with EtOH, MeCN, and acetone. There is no measurable leaching of MV^{2+} into solvents under these conditions.

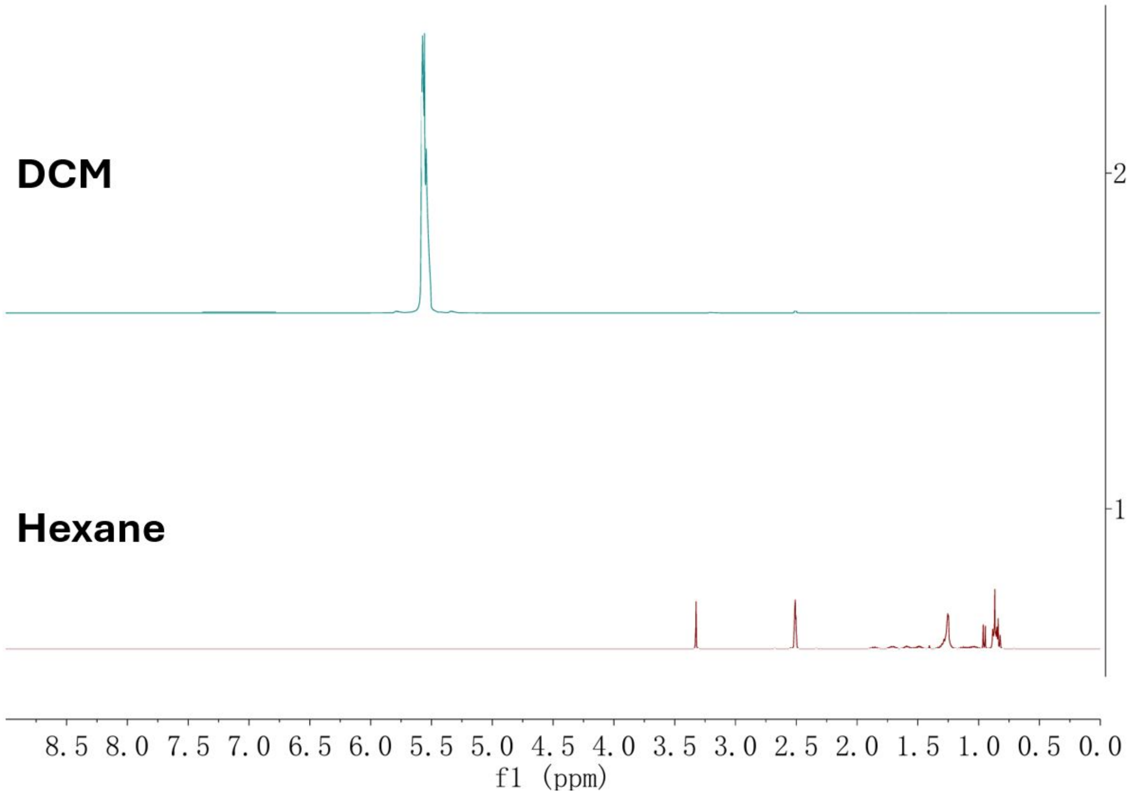

Figure S7. Solvent-stability test of $MVPb_2I_6$. The supernatant was extracted and added to DMSO-d_6 to run ^1H NMR of the supernatant after contacting $MVPb_2I_6$ with hexane and DCM. There is no measurable leaching of MV^{2+} into solvents under these conditions.

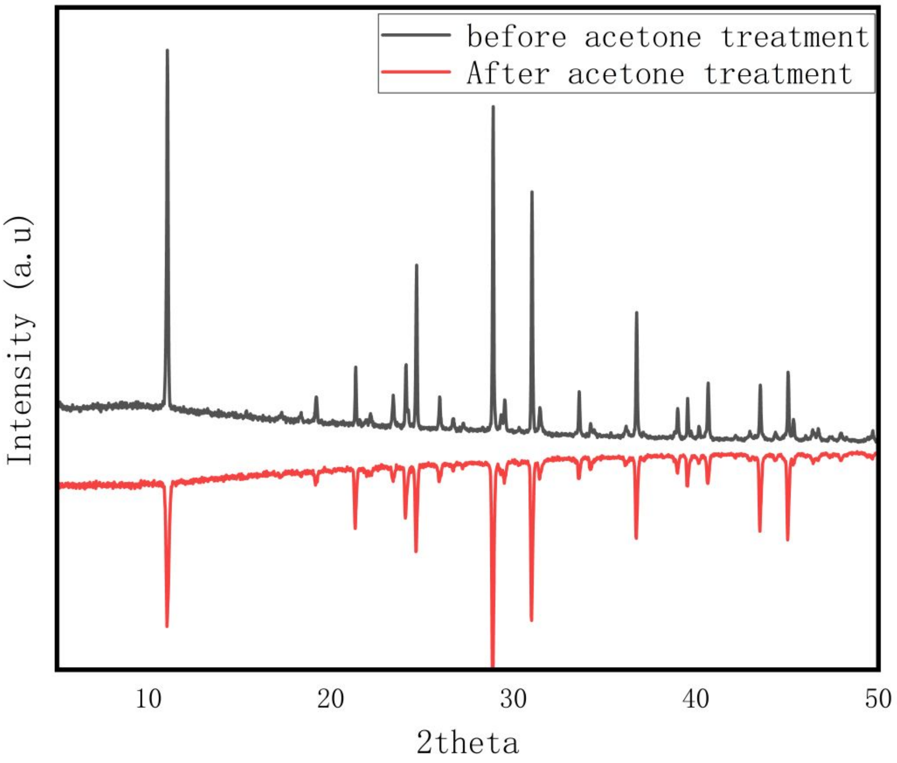

Figure S8. PXRD of $MVPb_2I_6$ before and after acetone treatment.

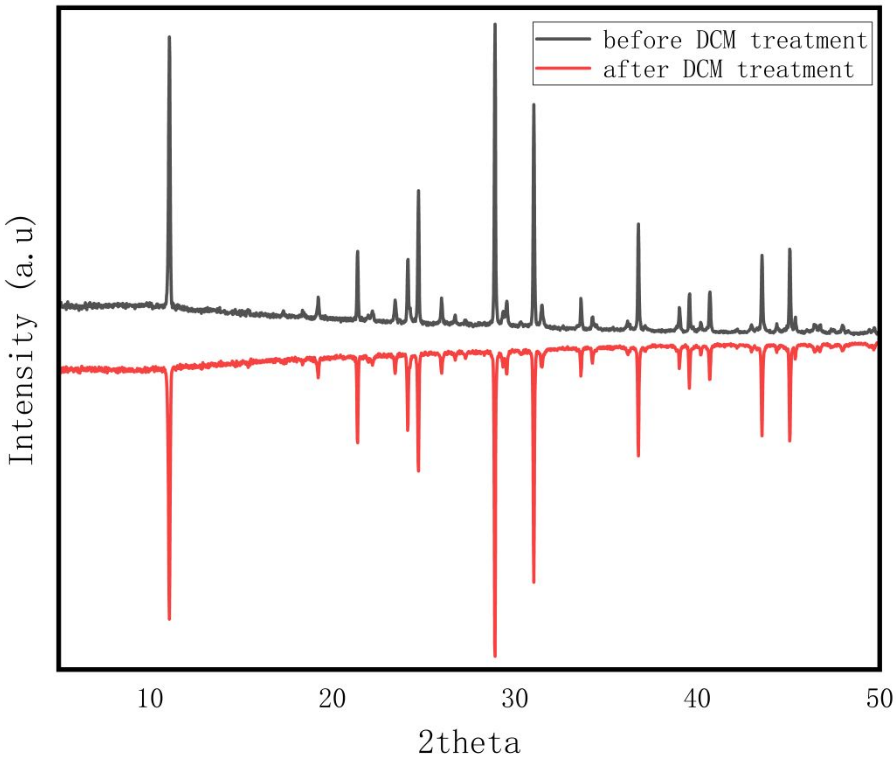

Figure S9. PXRD of $MVPb_2I_6$ before and after DCM treatment.

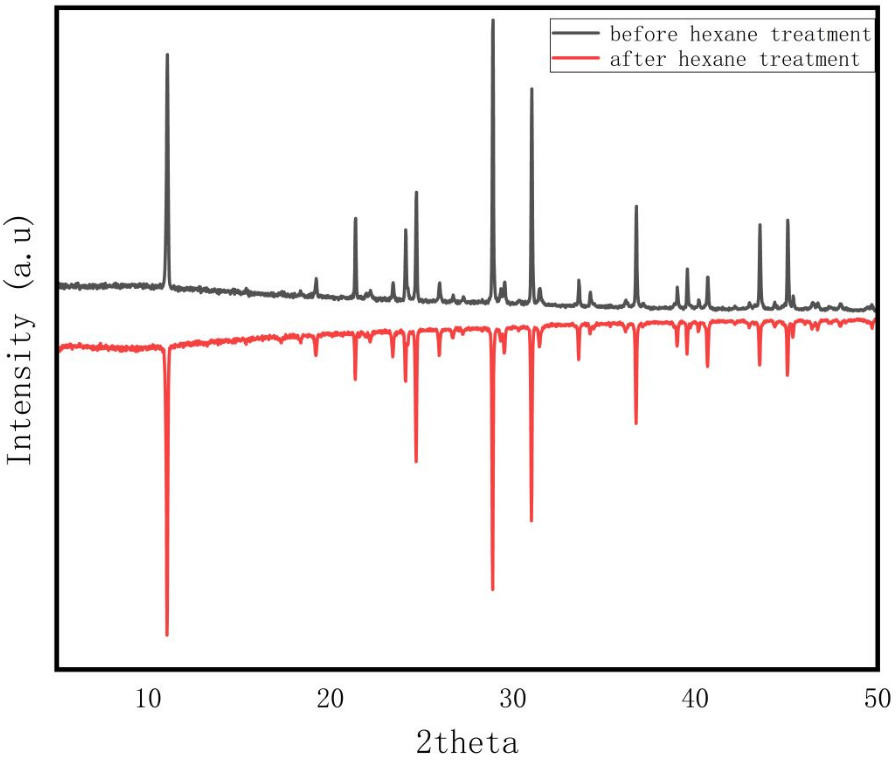

Figure S10. PXRD of $MVPb_2I_6$ before and after hexane treatment.

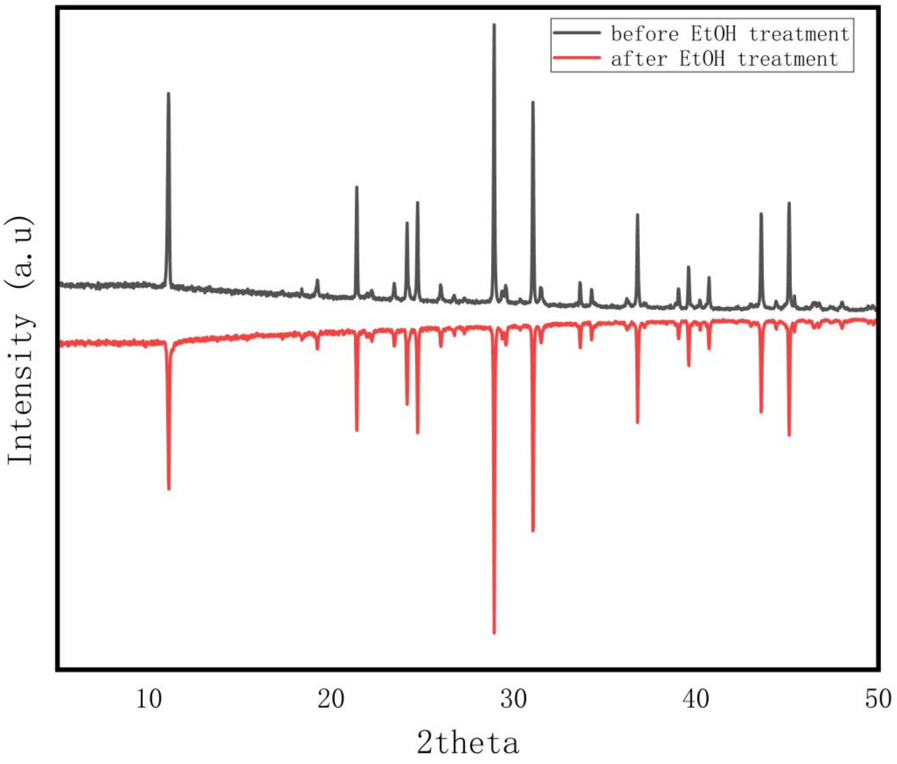

Figure S11. PXRD of $MVPb_2I_6$ before and after EtOH treatment.

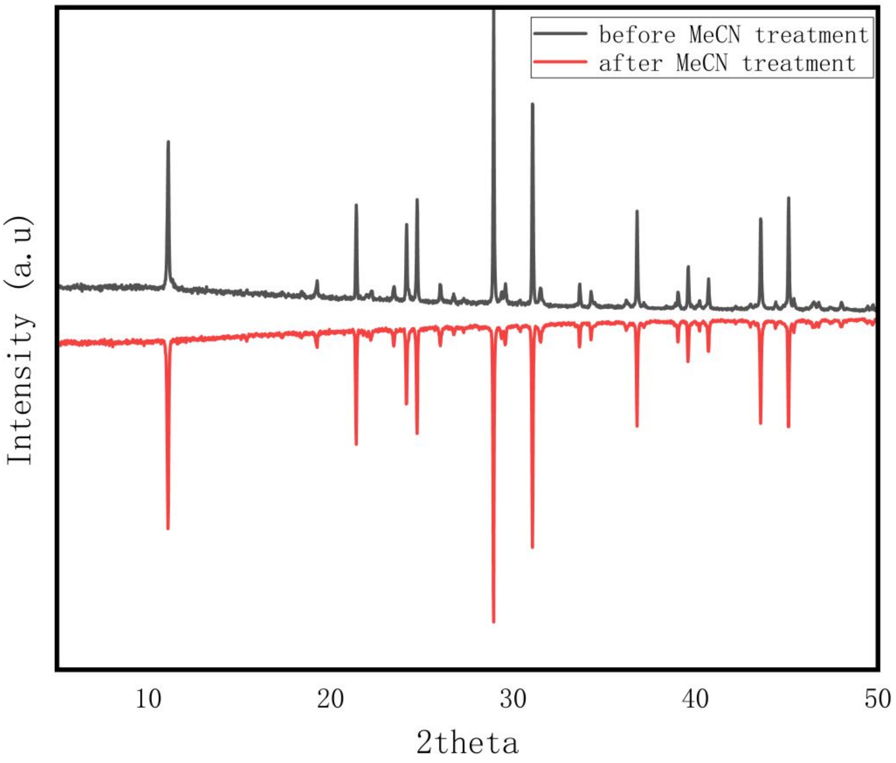

Figure S12. PXRD of $MVPb_2I_6$ before and after MeCN treatment.

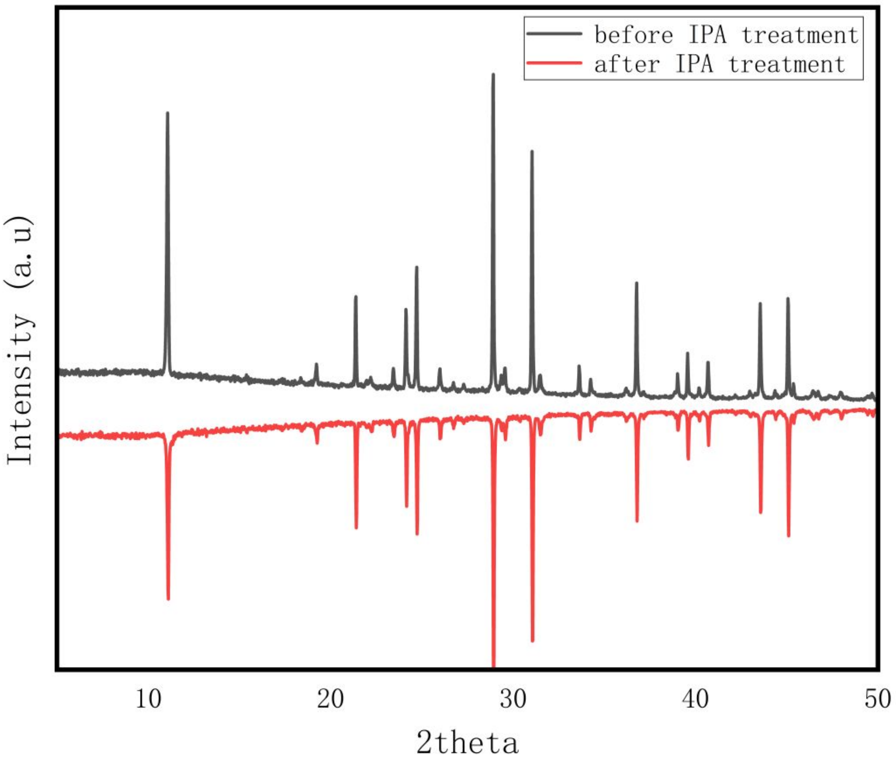

Figure S13. PXRD of $MVPb_2I_6$ before and after IPA treatment.

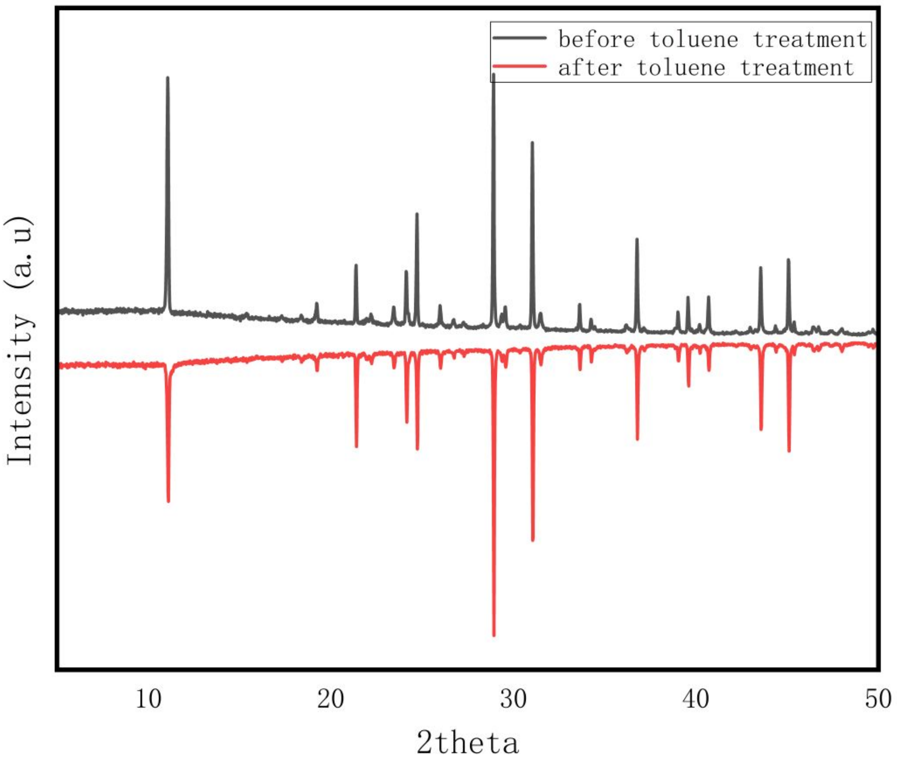

Figure S14. PXRD of $MVPb_2I_6$ before and after toluene treatment.

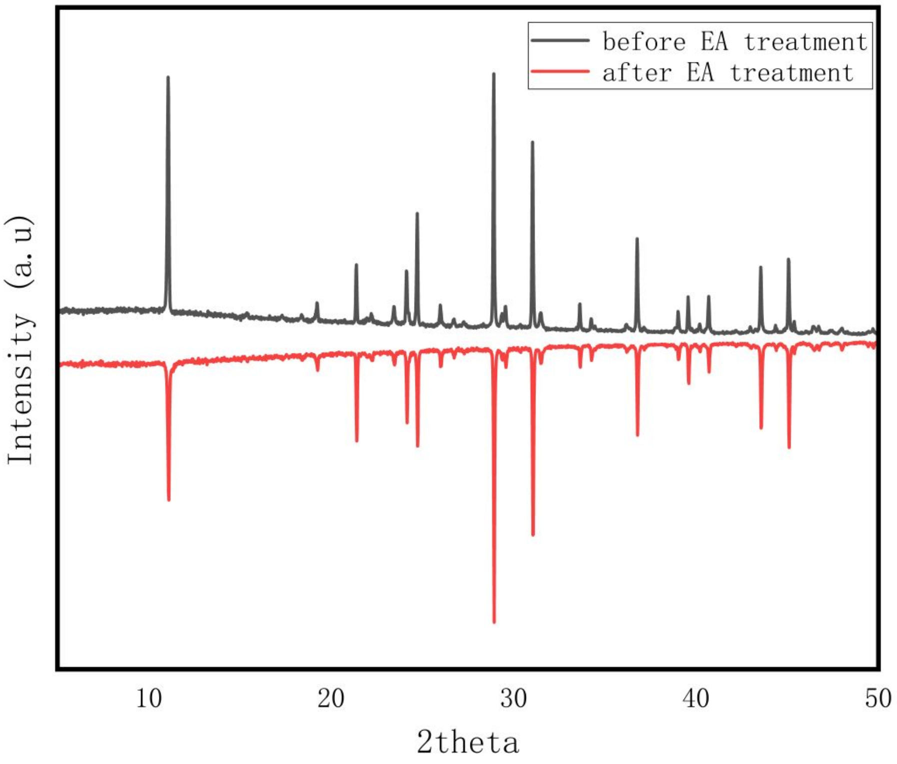

Figure S15. PXRD of $MVPb_2I_6$ before and after ethyl acetate treatment.

9. Mechanistic investigations regarding the reaction of 4-acetylpyridine by varying concentrations of acetic acid.

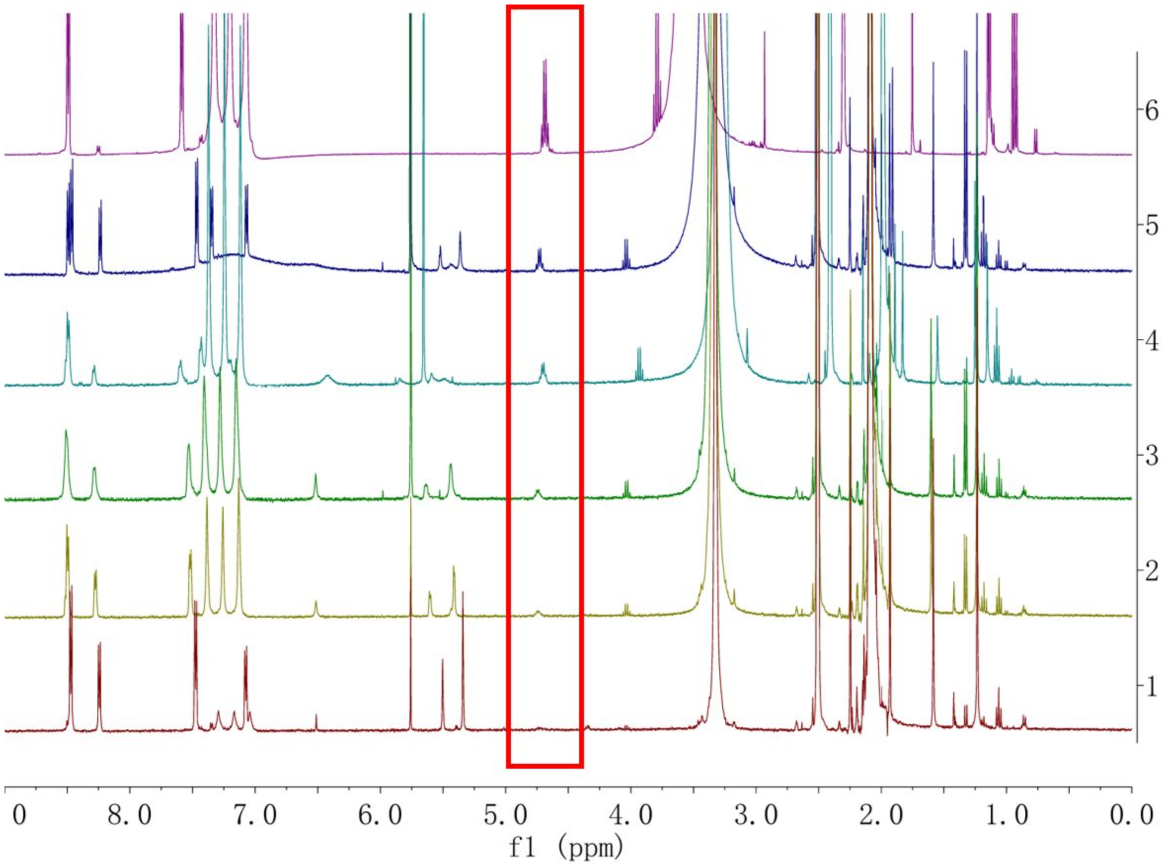

Figure S16. 1H NMR spectra (400 MHz, DMSO-d_6, 298 K) of 4-acetylpyridine photocatalytic reduction products under varying equivalents of acetic acid (AcOH: 10, 8, 6, 4, 2, 0 equiv, top → bottom). Operating the identical photocatalytic runs (10 → 0 eq acetic acid), the alcohol-diagnostic quartet at δ ~ 4.8 ppm decreases while the pinacol-diagnostic at δ ~ 5.5/5.3 increases. In the aromatic region, alcohol (δ ~ 8.5/7.3) and pinacol [meso (δ ~ 8.5/7.5) + dl (δ ~ 8.3/7.1)] overlap to give the apparent multi-peak pattern. [5,10–15]

While direct mechanistic data were not obtained in our current study, our rationale is informed by prior electrochemical investigations.[10] In particular, a detailed kinetic analysis by Saveant et al. demonstrated that the electrochemical reduction of 4-acetylpyridine exhibits a formal second-order dependence on proton concentration, as inferred from the pH-dependence of the half-wave potential. Based on these results, they proposed a three-step mechanism: (1) rapid protonation of the pyridyl ketone, (2) a concerted two-electron, one-proton reduction to generate a stabilized carbanion intermediate, and (3) a subsequent protonation to yield the alcohol product. In light of this established pathway, we hypothesize that the photoelectrochemical reaction under our conditions proceeds through an analogous sequence. We believe the mechanistic assignment is consistent with prior literature and offers a plausible explanation for the observed trends.

^{1}H and ^{13}C NMR spectra

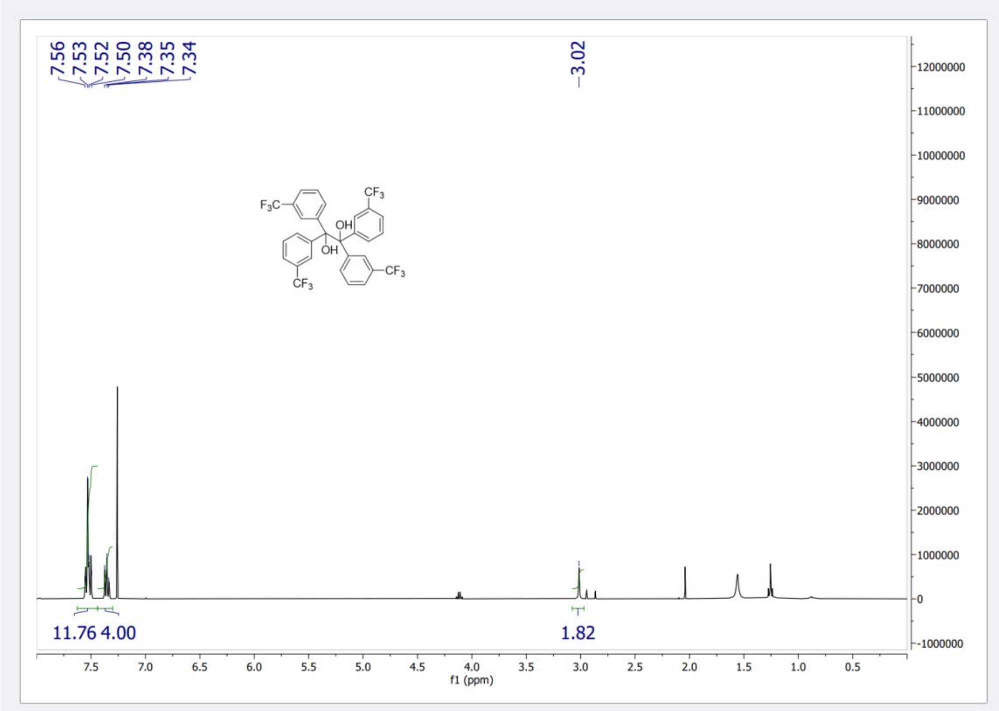

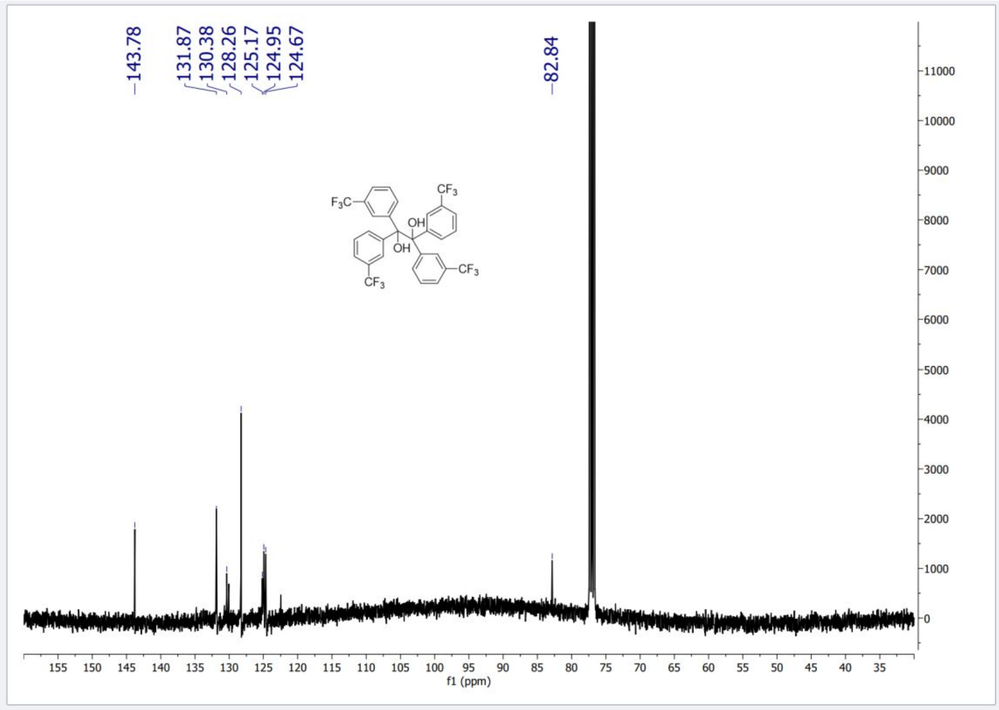

Figure S17. ^{1}H (400 MHz) and ^{13}C (125 MHz) NMR spectra of 1,1,2,2-tetrakis(3-(trifluoromethyl)phenyl)ethane-1,2-diol **2h** ($CDCl_3$)

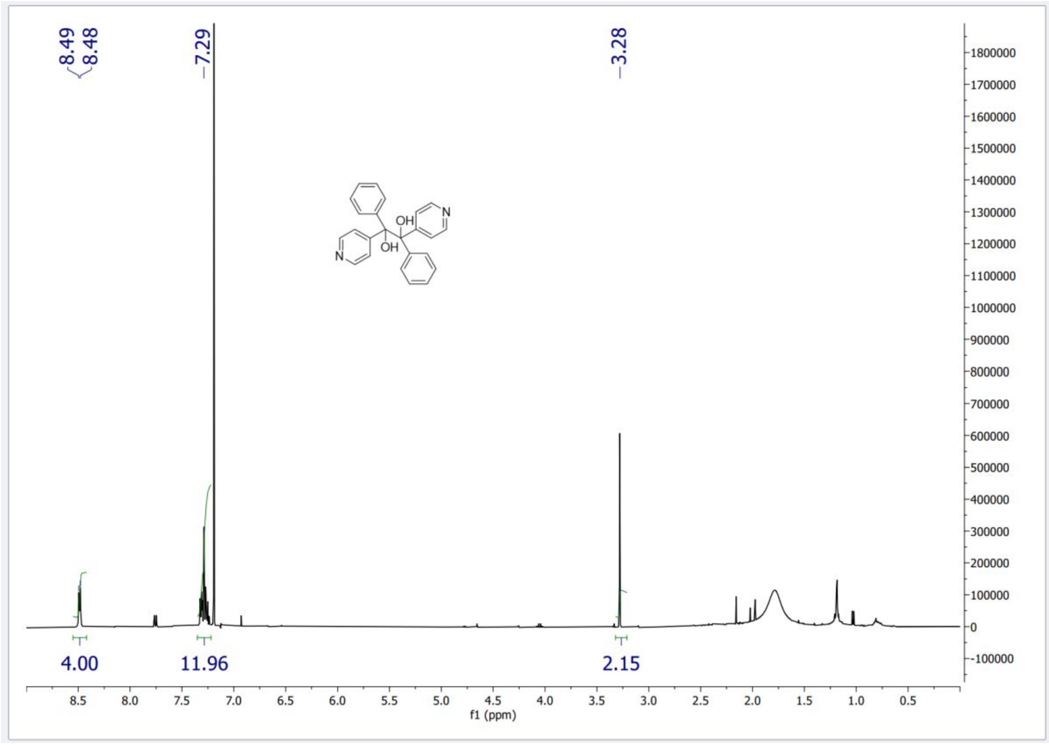

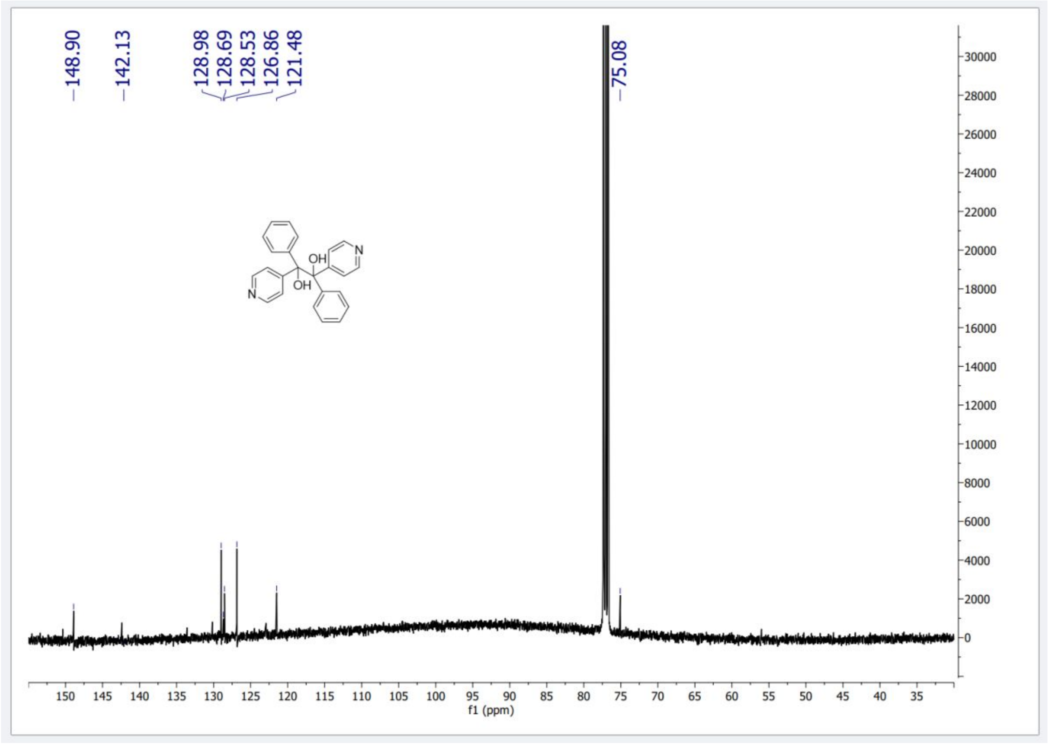

Figure S18. ^{1}H (400 MHz) and ^{13}C (125 MHZ) NMR spectra of 1,2-diphenyl-1,2-di(pyridin-4-yl)ethane-1,2-diol **2i.**

Reference:

(1) Lyu, R.; Cui, Z.; Elgin, J.; Co, A. C.; Wu, Y. Photoelectrochemistry of Methylviologen Lead Iodide: Achieving Stability inside a Polar Solvent. *J. Phys. Chem. C* **2023**, *127* (32), 15852–15860. https://doi.org/10.1021/acs.jpcc.3c04054.

(2) Tang, Z.; Guloy, A. M. A Methylviologen Lead(II) Iodide: Novel $[PbI_3^-]_\infty$ Chains with Mixed Octahedral and Trigonal Prismatic Coordination. *J. Am. Chem. Soc.* **1999**, *121* (2), 452–453. https://doi.org/10.1021/ja982702i.

(3) Wang, S.; Mitzi, D. B.; Feild, C. A.; Guloy, A. Synthesis and Characterization of [NH2C(I):NH2]3MI5 (M = Sn, Pb): Stereochemical Activity in Divalent Tin and Lead Halides Containing Single .Ltbbrac.110.Rtbbrac. Perovskite Sheets. *J. Am. Chem. Soc.* **1995**, *117* (19), 5297–5302. https://doi.org/10.1021/ja00124a012.

(4) Nakajima, M.; Fava, E.; Loescher, S.; Jiang, Z.; Rueping, M. Photoredox-Catalyzed Reductive Coupling of Aldehydes, Ketones, and Imines with Visible Light. *Angew Chem Int Ed* **2015**, *54* (30), 8828–8832. https://doi.org/10.1002/anie.201501556.

(5) Shen, Q.; Cao, K.; Chen, X.; Li, X.; Zhang, N.; Miao, Y.-B.; Li, J. Photo-Catalyst-Free Photomediated Pinacol Coupling of Ketones/Aldehydes by Formate at Room Temperature. *Green Chem.* **2023**, *25* (23), 9665–9671. https://doi.org/10.1039/D3GC01522J.

(6) Qiu, Z.; Pham, H. D. M.; Li, J.; Li, C.-C.; Castillo-Pazos, D. J.; Khaliullin, R. Z.; Li, C.-J. Light-Enabled Metal-Free Pinacol Coupling by Hydrazine. *Chem. Sci.* **2019**, *10* (47), 10937–10943. https://doi.org/10.1039/C9SC03737C.

(7) Guo, S.; Jiang, Y.; Yang, J.; Lu, G.; Liu, M.; Liu, W.; Cai, H. Chemo-Selective Electrochemical Pinacol Coupling of Aldehydes and Ketones Using $TMSN_3$ as a Promoter. *J. Org. Chem.* **2025**, *90* (6), 2139–2147. https://doi.org/10.1021/acs.joc.4c02147.

(8) Kuciński, K.; Hreczycho, G. Lithium Triethylborohydride as Catalyst for Solvent-Free Hydroboration of Aldehydes and Ketones. *Green Chem.* **2019**, *21* (8), 1912–1915. https://doi.org/10.1039/C9GC00216B.

(9) Rivero, A. R.; Kim, B.-S.; Walsh, P. J. Palladium-Catalyzed Benzylic Arylation of Pyridylmethyl Silyl Ethers: One-Pot Synthesis of Aryl(Pyridyl)Methanols. *Org. Lett.* **2016**, *18* (7), 1590–1593. https://doi.org/10.1021/acs.orglett.6b00450.

(10) Koster, K. Heterogeneous Catalysis and Electrosorption Phenomena in the Cathodic Reduction of Acetylpyridines Part II. Electrode Kinetic Investigations. *Journal of Electroanalytical Chemistry* **1983**, *157* (1), 89–111. https://doi.org/10.1016/0022-0728(83)80372-6.
